# Database Analysis of Depression and Anxiety in a Community Sample—Response to a Micronutrient Intervention

**DOI:** 10.3390/nu10020152

**Published:** 2018-01-30

**Authors:** Samantha M. Kimball, Naghmeh Mirhosseini, Julia Rucklidge

**Affiliations:** 1 Pure North S’Energy Foundation, Calgary, AB T2R 0C5, Canada; Naghmeh.Mirhosseini@purenorth.ca; 2 Department of Natural and Mathematical Sciences, St. Mary’s University, Calgary, AB T2X 1Z4, Canada; 3 Department of Psychology, University of Canterbury, Christchurch 8140, New Zealand; Julia.rucklidge@canterbury.ac.nz

**Keywords:** depression, anxiety, vitamin D, nutritional supplements, multivitamin/multimineral, mental health

## Abstract

Background: Depression and anxiety are common mental health concerns worldwide. Broad-spectrum multi-vitamin/mineral approaches have been found to alleviate a number of psychiatric symptoms. We investigated the effects of a nutrient intervention program, which includes optimizing vitamin D levels, on depression and anxiety outcomes from community-based program. Methods: We evaluated self-reported health measures of depression and anxiety collected as part of a community-based program focused on optimizing overall health through nutritional supplementation, education and lifestyle advice. Results: Data were collected from 16,020 participants, with measures including European Quality of Life Five Dimensions (EQ-5D) and Targeted Symptoms List (TSL) providing self-reported depression and anxiety. More than 56% of participants were identified as having elevated levels of depression and anxiety at baseline as reported on the EQ-5D. After one year in the program, 49.2% (*n* = 7878) of participants who reported any level of depression or anxiety at baseline reported improvement at follow-up. Of those who reported severe/extreme depression at baseline (*n* = 829), 97.2% reported improvement after one year. Regression analyses revealed a significant association of improvement in depression and anxiety with higher vitamin D status (>100 nmol/L) and more strenuous physical activity. Conclusion: Overall, people from the general population who suffer from mood and anxiety problems may benefit from improved nutritional status achieved with nutritional supplements.

## 1. Introduction

The World Health Organization estimates that 350 million people worldwide are affected by depression and anxiety. The Global Burden of Disease Study in 2010 identified depressive disorders as a leading cause of burden and responsible for 3.8% of global Disability Adjusted Life Years (DALY) [1]. As a contributor to DALY, depression is expected to be the second highest concern for all ages by 2020 [2]. In Canada, it is estimated that over 6.7 million people live with a mental health problem (in comparison with 3.5 million with type 2 diabetes mellitus) [3]. Economic costs of mental health problems are estimated to be at least $50 billion per year [3]. With current medications purported to only have a modest benefit [4], few new psychiatric drugs on the horizon, and some studies suggesting medication can do more harm than good [5,6,7,8], there are calls to revisit the impact that nutrition can have on mental health [4,9].

Biologically, nutrition is interconnected with depression through hormonal, neurotransmitter and signalling pathways in the gut that modulate brain functions such as appetite, sleep, reward mechanisms, cognitive function and mood [10]. All biochemical pathways require vitamins and minerals as co-factors for proper enzyme function and insufficient nutrient levels can negatively impact a wide range of metabolic processes. There are a number of different nutrients involved in pathways relevant to mental disorders and brain function [11].

Many nutrients have been investigated individually, including: B vitamins, vitamin D, choline, iron, zinc, magnesium, S-adenosyl methionine, amino acids such as taurine and probiotics. For the most part, studies of single nutrient treatments for mood disorders such as vitamin D report only modest symptom improvement or null results [12]. The evidence for B vitamins is stronger, such that a systematic review reported sufficient evidence to support the use of combination high dose B vitamins in the treatment of stress and anxiety in the general population [13]. Overall, no single nutrient stands out as a treatment for mental health problems. However, a broad-spectrum multivitamin and multimineral approach may alleviate symptoms in a number of psychiatric ailments.

Nutrition and dietary patterns have been linked with depression onset, and the maintenance and severity of symptoms [14,15,16,17]. Furthermore, several trials have demonstrated improvements in not only depression, but anxiety and stress response through supplementation with multivitamin/multimineral formulas and B complex vitamins [18,19,20,21,22,23]. In fact, the International Society for Nutritional Psychiatry Research has issued a consensus statement that “nutrition and nutraceuticals should now be considered as mainstream elements of psychiatric practice, with research, education, policy and health promotion reflecting this new paradigm [24]”. A full range of vitamins and minerals are obligatory cofactors involved in the production of neurotransmitters. For example, in the production of serotonin, the major neurotransmitter involved in depressive disorders, there are requirements for vitamin B6, vitamin D, iron, copper and molybdenum.

A multivitamin/mineral (MVM), in which many ingredients were given at levels higher than recommended dietary allowance (RDA) but generally lower than the upper level (UL), has been shown to improve depressive symptoms in bipolar individuals [25,26,27,28,29]. A MVM was also found to significantly reduce stress and anxiety following a series of earthquakes and a flood [22,29,30,31]. Despite enrolling participants who were not clinically depressed, two clinical trials have found that micronutrient supplementation improved symptoms of depression [32,33]. Collectively, the evidence suggests that a spectrum of nutrients may be effective for reducing and preventing depression, stress and anxiety. We hypothesized that optimal nutrient status, as measured by serum 25-hydroxyvitamin D (25(OH)D) levels above 100 nmol/L [34] and serum vitamin B12 above 450 pmol/L [35], would have a protective effect against depression and anxiety and that supplementation to achieve “optimal” nutrient status would improve depression and anxiety.

In the present study, we characterize the prevalence and severity of symptoms of depression and anxiety in participants in a community-based prevention program, before and after one year of exposure to nutritional supplements (a combination of vitamin D3, MVM and other nutrients), to determine if there was a benefit of supplementation with a broad spectrum of nutrients in a comparison sample. We further investigated the impact of supplementation in a subgroup of participants who self-reported extreme or severe depression and anxiety at entry to the program.

## 2. Materials and Methods

### 2.1. Intervention

The Pure North S’Energy Foundation (Pure North) is a not-for-profit wellness program focused on the prevention of chronic diseases including mental illness, type 2 diabetes mellitus, cardiovascular disease and cancer. The Pure North program offers lifestyle advice, education and nutritional supplements to its participants. Dietary advice typically includes aspects of the DASH diet developed to lower blood pressure without medication when appropriate for physical health outcomes, proper food portion education, encouraging plenty of fruit and vegetables (5–10 servings/day) and foods rich in omega-3 fatty acids, restriction of added sugar to less than 25 g/day and sodium to less than 2300 mg/day. Other lifestyle advice consists of proper sleep hygiene, smoking cessation, at least 150 min of physical activity each week and moderate alcohol consumption.

Supplement recommendations are based on analysis of each participant’s biometric measurements (including BMI), blood results and clinical intake data. Health care professionals review and explain blood work results with the participant and, based on their clinical knowledge and nutrient expertise, make recommendations accordingly. Each participant is treated as an individual and a treatment plan developed to meet that individual’s nutrient requirements. All participants are encouraged to achieve a 25(OH)D level of at least 100 nmol/L and vitamin D dosages were adjusted accordingly for the individual.

The core supplements provided to everyone are in daily packets, Vitality Packs, and contain: the multivitamin and multimineral formula (Vital 2 Platinum; Table S1), Omega-3 fatty acids (400 mg EPA and 200 mg DHA), Vitamin C (1000 mg), Vitamin B12 (5000 µg methylcobalamin), Probiotics (*Biffidobacterium and Lactobacillus strains*, 10 billion CFU) and Vitamin D3 drops. Vitamin D3 supplementation is individualized to target an optimal serum 25-hydroxyvitmainD (25(OH)D) concentration, above 100 nmol/L [36]. Doses of vitamin D3 are often in excess of the UL (4000 IU/d) and given under medical supervision [37]. Further supplements in addition to the Vitality Packs may be recommended based on individual requirements.

### 2.2. Assessments

Participants in the Pure North program are assessed at each visit to the clinic. Visits typically occur every 6–12 months to monitor lifestyle modification or disease condition change, and to adjust supplement doses if required. Any participant with obvious clinical depression or anxiety who requires closer monitoring or psychiatric medications is referred to their family physician. Assessments include biometrics (including blood pressure, height, weight, BMI, etc.), clinical intake (including medical history (such as pernicious anemia and *H. pylori* infections that might affect vitamin B12 absorption), current medications (including acid blockers and proton pump inhibitors that might affect vitamin B12 absorption), complaints and health goals), blood work (a number of biomarkers are measured including serum 25(OH)D, vitamin B12, arachidonic acid (AA) and eicosapentaenoic acid (EPA)), and completion of a questionnaire (including demographic data and self-reported health assessments). All blood sample preparation and biochemical measurements were performed by Doctor’s Data Laboratory (St. Charles, IL, USA), a fully accredited laboratory. Serum 25(OH)D was measured using Liquid Chromatography and tandem Mass Spectrometry (LC/MS-MS) with the inter-assay CV of 2.4%. Serum vitamin B12 was measured on an automated analyser with a chemiluminescent immunoassay (Beckman Coulter) with a CV of 7.7%. AA and EPA were measured using gas chromatography.

For the present study, we utilized serum 25(OH)D and vitamin B12 concentrations, available measures of nutrient status. Low levels of vitamin D have previously been linked to mental health disorders, including mood disorders such as depression [38,39,40]. Vitamin D is of particular interest due to its various roles in the brain and in neurotransmitter synthesis [41] and the heightened prevalence of vitamin D deficiency (serum 25(OH)D <50 nmol/L) and mood disorders in Canada (more than 32% and nearly 13%, respectively) [42,43]. B vitamins are important for adrenal support, energy production and neurotransmitter synthesis [44] and vitamin B12 deficiency (<148 pmol/L) is common worldwide [45]. Serum vitamin B12 is a measurable indicator of B12 status. Unfortunately, we are not easily and reliably able to measure the status of all nutrients in the body.

### 2.3. Self-Reported Depression and Anxiety

The European Quality of Life Five Dimensions (EQ-5D) assessment is a standardised instrument used to measure health status in a wide range of health conditions and treatments. The EQ-5D is a widely used questionnaire for calculating quality-adjusted life years (QALYs) for assessing cost-effectiveness in healthcare. Participants complete the EQ-5D at each visit to the clinic. The EQ-5D reflects the impact of common mental health conditions, such as mild to moderate depression and anxiety, on daily function [36] and has been found to be sensitive enough to pick up improvements associated with treatment in patients with depression [46]. We utilized the EQ-5D to investigate self-reported depression and anxiety in a community sample.

In addition to the EQ-5D participants completed the Targeted Symptoms List (TSL)—a wellbeing assessment tool developed in-house. The TSL assesses 16 mental and physical health outcomes on a scale of 1–10 (1 = never through to 10 = always) with a focus on common health complaints including: anger, anxiety, confusion, coordination problems, depression, fatigue, headache, joint pain, loss of sense (smell/taste), memory loss, moodiness, muscle weakness, numbness in arms/legs, shakiness of hands, stomach problems (digestive), and unintentionally dropping things. In addition, the TSL mental health score (MHS) is a composite score of depression, anxiety, and moodiness. For the purposes of the present study, we have focused on 3 TSL scores: depression, anxiety and the calculated MHS.

Depression and anxiety were measured via self-report in the population at baseline, the time of their first interaction with the Pure North program with a closer look at participants who reported severe or extreme depression/anxiety on the EQ-5D. We further utilized blood markers of nutritional status, 25(OH)D and vitamin B12, to investigate whether the effects of the nutrition-based intervention on levels of reported depression, anxiety and MHS were associated with changes in these nutrients over time.

This study was an analysis conducted utilizing a database of participant information collected as part of a community program (secondary use of data). The protocol of current database analysis was approved by the Research Ethics Board at St. Mary’s University in Calgary, Alberta (File# 073FA2017). Informed consent was obtained from all individual participants included in the study to use their information to evaluate the program.

### 2.4. Statistical Analyses

Statistical analyses were completed using SPSS v23 (SPSS Inc., Chicago, IL, USA). Descriptive statistics are presented as mean ± standard deviation. Intent-to-treat analyses were performed; as such mean follow-up values were used for participants who had baseline measures but not follow-up measures (mean imputation method). Since the data were not normally distributed, non-parametric tests were applied. Wilcoxon Signed Rank Test (WSRT) was performed to evaluate changes in different parameters over one-year. Mann–Whitney U-test and Kruskal–Wallis tests were utilized to compare categorical groups of vitamin D and vitamin B12 status. Chi Square testing was performed to determine the association between levels of anxiety and depression and 25(OH)D (>100 nmol/L) and B12 status (>450 pmol/L). Binary Logistic Regression was performed to investigate the relationship between 25(OH)D and vitamin B12 with changes in mental health status over time. Body mass index (BMI), age, gender, tobacco use, alcohol use, physical activity, fruit and vegetable consumption, fish and tuna consumption, AA:EPA and inflammation (CRP ≥ 10 mg/L) were considered as cofactors in these analyses. Significance was defined as *p* < 0.05. For determining effect size (ES), Cohen’s test was conducted using STATA version 14. Cohen’s d was used to estimate effect sizes, with 0.2 indicating a small effect, 0.5 a medium effect and 0.8 a large effect.

## 3. Results

### 3.1. Baseline Demographics

Data were collected for 16,020 participants (60% female) who entered the program and completed an EQ-5D. The mean age was 54.2 ± 16.0 (range 18–95) years. Of these, 1971 (16%) self-reported mental health problems or previous diagnoses of depression, anxiety, stress, Attention-Deficit/Hyperactivity Disorder (ADHD), Post-Traumatic Stress Disorder (PTSD), Seasonal Affective Disorder (SAD), or other mental health disorders. There were 2123 (20%) who reported taking medications used to treat depression and anxiety, of which 45.0% reported both depression or anxiety and taking medication. At program entry, serum 25(OH)D level was not significantly different between participants taking medication (77.8 ± 27 nmol/L) and those not (77.9 ± 27 nmol/L). There were 3875 participants who reported hypertension, 1656 with diabetes, and 1642 cases of cancer (past or present). Vitality packs were provided for 96% of participants and equivalent supplements provided to the remaining 4% who did not take the vitality pack for various reasons (e.g., one of the supplements was deemed unnecessary or did not agree with the participant). In addition, 44% of participants were recommended extra nutrients based on individual need.

The body mass index (BMI) distribution was 35.0% normal (18.5–24.9), 35.7% overweight (25–29.9) and 27.7% obese (>30), similar to the measured BMIs for the Canadian population reported by Statistics Canada at 36.1%, 36.8% and 25.1%, respectively [47]. At program entry, 84.0% of participants were considered to have suboptimal vitamin D levels (25(OH)D < 100 nmol/L) with 14.7% being deficient (25(OH)D < 50 nmol/L). Suboptimal vitamin B12 levels, <450 pmol/L, were found in 75.5% of participants at baseline. Medical conditions that can influence vitamin B12 absorption were investigated. In the four cases of pernicious anemia, serum vitamin B12 levels increased from 214 to 1159 pmol/L, similar to response found in the rest of the population. There were three cases with *H. pylori* infection in whom serum vitamin B12 levels increased from 308 to 503 pmol/L. Twelve percent of participants were taking acid blockers, such as proton pump inhibitors, with no significant difference in serum vitamin B12 levels between participants taking (427 ± 1543 increased to 1049 ± 2662 pmol/L) and those not taking (421 ± 1047 increased to 995 ± 1264 pmol/L) acid blockers.

More than half of participants (56.2%) reported some level of depression or anxiety at the entry to the program (Table 1). Eighty-one per cent of participants who reported a history of depression or anxiety and 87% who reported taking medications for depression and anxiety, reported low levels of depression and anxiety at baseline (none to moderate). Five percent (5.2%) of the population reported severe or extreme depression at program entry, with more than half of those having reported a history of or taking medications for depression/anxiety (Table 1). After one year in the program, there was no significant difference in serum 25(OH)D levels between participants taking anxiety or depression-related medication (111 ± 31 nmol/L) and those not taking medications (112 ± 32 nmol/L). There was no significant difference in the proportion of participants taking medications for anxiety or depression (1.0%) in comparison with those not taking medications (1.3%).

The severity of depression/anxiety was also significantly correlated with several lifestyle characteristics (Table S2). Participants who reported severe or extreme depression/anxiety at program entry often consumed no fruit (*p* < 0.001), vegetables (*p* < 0.001), and fish (*p* = 0.03) or consumed fewer servings relative to those who were not depressed/anxious. Participants who used tobacco (*p* < 0.001), drank heavily (*p* < 0.001) and were less physically active (*p* < 0.001), were also likely to report severe or extreme depression/anxiety at program entry. Serum 25(OH)D levels were significantly lower in depressed/anxious participants who were less physically active (66.5 ± 28 nmol/L) compared to those who were more physically active (76 ± 24 nmol/L).

Because of the often cited ceiling effect found with the EQ-5D [48,49,50], we utilized a second measure of depression and anxiety from an in-house questionnaire, the TSL, which had been previously validated against both the EQ-5D and the SF-12 Health Survey. Reported depression/anxiety on the EQ-5D is compared with TSL scores in Table 2. The depression/anxiety aspect of the EQ-5D followed similar distribution patterns as TSL scores. Reported depression/anxiety on the EQ-5D had a significant correlation with the TSL anxiety (*r* = 0.66, *p* < 0.001), depression (*r* = 0.67, *p* < 0.001) and MHS (*r* = 0.71, *p* < 0.001). In addition, the TSL mental health score significantly correlated with the SF-12’s Mental Component Summary (MCS-12) score in a subset of participants who completed both (*n* = 2084, *r* = 0.68, *p* < 0.001; data not shown). These results support the use of the MHS to assess mental wellbeing and to investigate nutrient effects on depression and anxiety in this population.

### 3.2. Comparison between Baseline and One-Year Follow-Up

All participants at baseline were included in this intent-to-treat analysis (*n* = 16,020). There were 4159 participants who returned for their follow-up in the clinic within 6–18 months after the initial visit, average 12 months. A comparison of baseline measures between participants who persisted in the program (returned for follow-up in the specified time frame) and those that did not, found that Persisters were older, more were female, they had higher baseline 25(OH)D and vitamin B12 levels, lower AA:EPA and reported less depression (mean 3.04 vs. 3.73 on TSL) and anxiety (mean 3.36 vs. 4.04 on TSL) (Table 3).

After an average of one year, we examined levels of depression/anxiety reported on the EQ-5D at follow-up. Overall, 46.8% of participants did not report any change in their level of depression/anxiety, whereas 4.1% reported higher levels and 49.2% reported improvement (Table 4). Of those who reported severe/extreme depression at baseline [*n* = 577 (severe), *n* = 249 (extreme)], 97.2% reported improvement, 2.4% reported no change and 0.4% were worse after one year in the program (Chi-square, *p* < 0.05). For those who reported none/slight/moderate levels of depression or anxiety, 58.4% improved, 3.6% worsened and 38% did not report any change. Per protocol analyses revealed similar results to ITT (data available upon request).

We validated the use of TSL scores as a numeric scale by comparing baseline TSL scores between those who reported severe/extreme depression on the EQ-5D with those who reported none, slight or moderate levels. As expected, those who reported severe/extreme depression/anxiety on the EQ-5D had significantly higher mean TSL scores than the rest of the population for both depression (7.65 ± 2.4 vs. 3.4 ± 2.4, respectively) and anxiety (7.9 ± 2.3 vs. 3.7 ± 2.4, respectively). Effect sizes were used to compare the magnitude of change between baseline and follow-up. Effect sizes were much larger for those who started the nutrients in a severe/extreme depressed or anxious state (Table 5).

We examined the effect of the nutrient intervention program on changes in measured nutrients, serum 25(OH)D, vitamin B12 and AA:EPA in relation to baseline depression/anxiety level. Similar significant improvements in 25(OH)D, vitamin B12 and AA:EPA were found in both groups (severe/extreme vs. none/slight/moderate), without any reported side effects (Table 6). Per protocol analyses revealed similar results to ITT (data available upon request).

After one year in the program, more than 62% of participants had serum 25(OH)D levels above 100 nmol/L compared to 25% at baseline. Kimball et al. (2017) [37], assessing safety in the Pure North population, previously showed that serum 25(OH)D levels up to 300 nmol/L were found to be safe, without any evidence of toxicity. In the current study, following vitamin D supplementation and a significant increase in serum 25(OH)D levels over time, no participant obtained a level above 300 nmol/L and there were no cases of vitamin D toxicity.

To investigate whether the improvements in depression and anxiety were related to the nutrient supplementation program, we utilized serum 25(OH)D and vitamin B12 levels. We compared 25(OH)D and vitamin B12 levels between those participants who reported being severe/extreme depressed or anxious versus being none/slight/moderate depressed or anxious at baseline (Table 7). We compared the magnitude of the changes between baseline and one year in participants considered deficient with those who had reached optimal levels for vitamin D and B12 at follow-up, for both categories. Among those who were severe/extreme anxious or depressed, moderate effect sizes, ranging from 0.5 to 0.6, were found in participants who remained below optimal vitamin B12 levels (<450 pmol/L) and serum 25(OH)D levels (<100 nmol/L). In contrast, those participants who achieved optimal vitamin B12 and optimal 25(OH)D at follow up, experienced significant improvements in depression, anxiety and MHS on TSL with effect sizes in the range of 1.95 to 2.33. In participants with optimal vitamin B12 and serum 25(OH)D at baseline, the effect sizes for depression/anxiety outcomes were similar to effect sizes when either 25(OH)D levels or vitamin B12 levels were in the optimal range alone. Among those who were none/slight/moderate anxious or depressed at baseline, there was no effect of the program when participants had low vitamin B12 (<450 pmol/L) and 25(OH)D levels (<100 nmol/L) with small but significant ES in those with high vitamin B12 and 25(OH)D that ranged from 0.22 to 0.29. Effect sizes were similar but much larger in those who were in the severe/extreme group, with effect sizes that ranged from 1.95 to 2.33 in the high vitamin B12 and 25(OH)D groups (Table 7). Per protocol analyses revealed similar results to ITT (data available upon request).

The effect of the nutrients on mental health outcomes were assessed with a nested case control analysis. We selected 107 participants who were severely/extremely depressed/anxious (based on EQ-5D) but did not take any psychiatric medication (cases), and compared them with 214 severely/extremely depressed/anxious participants, matched based on age, sex and BMI, who took related psychiatric medication (controls). Following one year in program, nutrient levels significantly increased in both the case and control groups. Serum 25(OH)D levels were higher in participants who were not taking psychiatric medications in comparison with those taking medications at both baseline and follow-up. There was no significant difference between depressed/anxious participants taking vitamins and those taking both vitamins and medications (Table 8).

We additionally compared EQ-5D score improvements between depressed/anxious participants taking no medication (cases) and those taking medication (controls). Again, we did not find any significant difference between case and control groups (Table S3). In both groups, more than 90% showed improvement in their mental status and the rest remained unchanged, regardless of their related psychiatric medication status. No participants reported a worsened mental status. 

We further investigated the effects of nutrient status on mental health outcomes through logistic regression analyses. Participants taking anxiety- and/or depression-related medications were excluded from the regression analysis (*n* = 700). Improvements in serum 25(OH)D concentration, such that participants went from having suboptimal levels to optimal levels (≥100 nmol/L for 25(OH)D), were found to be associated with improvements in anxiety, depression and MHS (Table 9). In addition, physical activity significantly influenced anxiety and depression scores with moderate and strenuous physical activity related to larger improvements to MHS. Age also influenced anxiety, depression and MHS with younger participants showing larger improvements. Current tobacco use and inflammation negatively affected mental health status.

## 4. Discussion

This study found that participants in an intervention program aimed at chronic disease prevention that provides nutritional supplements showed significant improvement in self-reported depression and anxiety over the course of one year. This was a large database with over 16,000 participants who had completed the questionnaires, EQ-5D and the TSL, at program entry. At baseline, over half of these participants reported some level of depression and anxiety, suggesting that a large number of people suffer from subclinical mood disturbances. Severe and extreme depression and anxiety was reported in 5.2% of the population which is consistent with a prevalence of depression of 5.4% in Canadians [3,51].

Overall, significant improvements were found for depression and anxiety after one year in the program. This is notable given that the majority of the population reported only mild levels of depression and anxiety at baseline (effect size 0.21 and 0.22, respectively). More notable, over 90% of those who reported severe or extreme depression or anxiety at program entry reported improvement at one year (effect size 1.85 and 1.94, respectively).

Vitamin D deficiency was highly prevalent among severely/extremely depressed/anxious participants and achieving physiological serum 25(OH)D concentrations (above 100 nmol/L) was found to have a significant influence on mental health improvements in the context of the rest of the program, particularly for depression.

All participants received multiple nutritional supplements including the Vital 2 Platinum multivitamin/mineral supplement, vitamin B12, omega 3 and vitamin D_3_ supplementation. However, achieving serum 25(OH)D concentrations above 100 nmol/L and vitamin B12 levels above 450 pmol/L, safely [37], positively influenced depression and anxiety outcomes. The results suggest that nutrient intervention impacts depression to a greater extent than anxiety, but both were positively influenced. Others have found that vitamin D_3_ supplementation has led to improvements in depression [40,52,53,54,55,56,57], yet others have not [58,59,60]. A meta-analysis demonstrated the importance of dose, frequency and achieving measurable increases in 25(OH)D concentrations in being able to detect favourable depression outcomes [61]. Our results support those of Kaplan et al. who demonstrated that vitamin B complex and multivitamin/mineral formulas have resulted in greater reductions in stress and anxiety compared with vitamin D_3_ supplementation alone [29]. A systematic review of the evidence demonstrates a significant number of trials that overall provide sufficient support for the use of B vitamins in the treatment of stress and anxiety in the general population, the benefit of which may be augmented with a broad array of minerals [13]. It is hypothesized that assuring all of the necessary cofactors for all metabolic processes improves mental resilience to stress and thus improves one’s ability to cope. Given that the clinical intervention involves vitamin D_3_ in combination with MVM and vitamin B12, among other nutrients, the authors posit that, where vitamin D_3_ was found to be a significant predictor of improvements, the magnitude was dependent upon an optimal background of other nutrients. While optimal vitamin D levels are necessary for nearly every cell, it is but one component needed for proper functioning of all metabolic systems of the body. Moreover, the absorption of certain nutrients such as vitamin B12 may be affected by different disease conditions (pernicious anemia, and *H. pylori* infection) or medications such as acid blockers or psychiatric medications that may inhibit vitamin D absorption. These may explain the discrepant results from other studies involving vitamin D_3_ or vitamin B12 as a stand-alone treatment.

More strenuous physical activity was found to be associated with improved mental health status. There is growing evidence that physical activity has protective effects not only on physical health but also on mental health, particularly depression and anxiety. Engaging in regular physical activity led to fewer depression and anxiety symptoms than did being inactive, which may also be related to vitamin D synthesis via sun exposure [62,63,64]. The anti-inflammatory effect of omega 3 have been successfully linked to preventing or treating depression [65,66], and B vitamins appears to treat anxiety, depression and stress [13,67], although we found the predominant effect for vitamin D.

The strengths of the present study include the large community-based population. The authors are unaware of any other study of a clinical intervention with as many participants as included here. Further, the population was not limited to clinically depressed individuals and found beneficial effects for the population in general.

The main limitations of this study are the lack of details of the other treatment options participants engaged in outside of the program (e.g., mediation or counselling). The data analysed here were collected as part of a clinical intervention, a real life program not specifically designed for research; hence, we were not able to ensure compliance and a high drop-out rate was expected. We used intent-to-treat analysis and found results compatible with the results of per protocol analysis. Following program entry, participants were provided with a standard package of multiple nutrients (multivitamin/mineral, vitamin D_3_, omega-3 fatty acids, vitamin B12, vitamin C and probiotics). In addition, nearly half of the participants were recommended additional supplements based upon individual requirements—for example, magnesium may have been recommended to an individual experiencing leg cramps or constipation. Vitamin D_3_ supplementation in the program was targeted at achieving a serum 25(OH)D concentration above 100 nmol/L rather than a specific dose of vitamin D_3_, but the rest of the supplements were provided at the standard dose. Because the data are from a voluntary health and wellness program, albeit a clinical intervention, there is likely to be a selection bias. Further, due to the nature of the study we were not able to measure compliance to the program, but the results do reflect real-life response. Small but significant differences were detected in a baseline comparison between those who persisted in the program and those who did not; there was a significantly higher proportion (6.0%) of severe/extreme depressed/anxious at baseline who dropped-out of the program in comparison with those who persisted to one year (2.7%). In addition, the measurement of depression and anxiety was through a standardized questionnaire rather than a clinical assessment. The results presented here represent a real-life scenario rather than a clinical study.

Currently, the front line form of treatment for depression is psychopharmaceutical, but there is growing interest in exploring the role of nutrients. Nutritional status plays an important role in mental health, and poor nutrition may contribute to the pathogenesis of mental illness. Nutritional deficiencies could have an influence on brain structure and function, including mood, anxiety and depression. We found depression and anxiety were commonly reported in the participants of this clinical intervention. This analysis showed that improvements in depression and anxiety were associated with achieving serum 25(OH)D concentrations of at least 100 nmol/L. A nutrient-based program aimed at achieving optimal nutrition may provide a safe, simple and economical means of supporting mental health.

## 5. Conclusions

Overall, this analysis of a large database sample (*n* = 16,020), in which over half of the population reported some level of depression and anxiety, confirms the high rate of people in the general population who suffer from mood and anxiety problems. The current study suggests that general mood may benefit from improved nutritional status. Nutritional status plays an important role in mental health, and poor nutrition may contribute to the pathogenesis of mental illness. Broad spectrum supplements with a focus on optimizing vitamin D status may provide a new paradigm for treatment and prevention of mental illness.

## Figures and Tables

**Table 1:** Baseline demographics and self-reported levels of anxiety and depression on European Quality of Life Five Dimensions (EQ-5D).

Level of Anxiety or Depression Reported on EQ-5D	Total (%)	Female (%)	% with Reported History of Depression/Anxiety	% with Reported Medication Use for Depression/Anxiety
None	7014 (43.8)	3951 (56.3)	231 (4.4)	418 (9.3)
Slight	5417 (33.8)	3451 (63.7)	598 (14.3)	702 (20.3)
Moderate	2763 (17.2)	1675 (60.6)	772 (34.0)	718 (38.8)
Severe	577 (3.6)	323 (56.0)	243 (49.5)	184 (46.5)
Extreme	249 (1.6)	156 (62.7)	127 (62.3)	101 (61.2)
Total	16,020 (100)	9556 (59.7)	1971 (16)	2123 (20.5)

**Table 2:** Baseline Comparison of European Quality of Life Five Dimensions (EQ-5D-Rated) Level of Depression/Anxiety to Targeted Symptoms List (TSL) Scale Scores for Depression and Anxiety.

EQ-5D Depression/Anxiety	Mean TSL Depression Score	* *p*-Value	Mean TSL Anxiety Score	* *p*-Value	Mean TSL Mental Health Score	* *p*-Value
None	1.95 ± 1.5	<0.001	2.21 ± 1.6	<0.001	2.11 ± 1.3	<0.001
Slight	4.00 ± 2.0		4.33 ± 2.0		3.98 ± 1.7	
Moderate	5.84 ± 2.3		6.25 ± 2.2		5.68 ± 1.8	
Severe	7.50 ± 2.3		7.69 ± 2.2		7.11 ± 1.8	
Extreme	8.00 ± 2.6		8.31 ± 2.4		7.57 ± 2.2	

* Kruskal–Wallis test (between levels comparison).

**Table 3:** Baseline comparison of Participants who stay in the program for one year (Persisters) to those that had a baseline visit only (Drop-outs).

Variable	Persisters (*n* = 4159)	Drop-outs (*n* = 11,861)	*p* Value
Age (years)	62.9 ± 11.9	51.1 ± 16.7	<0.001 *
% Female	63.4	58.3	<0.001 ^#^
BMI	27.6 ± 5.5	27.5 ± 5.8	0.6
25(OH)D (nmol/L)	85 ± 31	73 ± 27	<0.001 *
Vitamin B12 (pmol/L)	454 ± 325	358 ± 286	<0.001 *
AA:EPA	13.6 ± 7.1	18.3 ± 7.7	<0.001 *
EQ-5D Depression and Anxiety, *n* (%):			
- None - Slight - Moderate - Severe - Extreme	2130 (51.2) 1329 (32) 585 (14.1) 84 (2) 31 (0.7)	4884 (41.2) 4088 (34.5) 2178 (18.4) 493 (4.2) 218 (1.8)	<0.001 ^#^
TSL mental health score	3.04 ± 2.0	3.75 ± 2.2	<0.001 *
TSL anxiety	3.36 ± 2.3	4.04 ± 2.6	<0.001 *
TSL depression	3.04 ± 2.3	3.73 ± 2.6	<0.001 *

* *p* value < 0.05 (Mann–Whitney U-test), ^#^ *p* value < 0.05 (Chi square test).

**Table 4:** Change in Reported Level of Depression and Anxiety at One Year.

Baseline Level of Depression/Anxiety	Change at One Year Number (%)			
	Worse	Improve	No Change	Total
None	430 (6.1)	0	6584 (93.9)	7014
Slight	177 (3.3)	4569 (84.3)	671 (12.4)	5417
Moderate	40 (1.4)	2511 (90.9)	212 (7.7)	2763
Severe	4 (0.7)	552 (95.7)	21 (3.6)	577
Extreme	0	246 (98.8)	3 (1.2)	249
Total	651 (4.1)	7878 (49.2)	7491 (46.8)	16,020
Chi-Square	*p* < 0.001			

**Table 5:** Effect Sizes (ES) * for Targeted Symptoms List (TSL) Levels of Depression, Anxiety and Mental Health Score.

TSL Score	EQ-5D “Severe/Extreme” for Depression/Anxiety	EQ-5D “None/Slight/Moderate” for Depression/Anxiety
ES for Depression	1.85 (1.71–1.99)	0.21 (0.18–0.23)
ES for Anxiety	1.94 (1.80–2.08)	0.22 (0.19–0.24)
ES for MHS	2.07 (1.93–2.22)	0.27 (0.24–0.30)
N	563	11,033

* Cohen’s d (with 95% confidence intervals) measure of effect sizes: 0.2 is small, 0.5 is medium, and 0.8 is large MHS = Mental Health Score.

**Table 6:** Comparison of biomarkers of nutrition with levels of depression and anxiety.

Parameter	Depressed/Anxious (Severe/Extreme) (*n* = 826)		Less Depressed/Anxious (None/Slight/Moderate) (*n* = 15,194)	
	Baseline	One Year	Baseline	One Year
Age (years)	47.2 ± 16	---	54.6 ± 16	---
BMI	27.6 ± 6.6	27.7 ± 2.9	27.5 ± 5.6	27.6 ± 3.0
Serum 25(OH)D (nmol/L)	71 ± 28	111 ± 16 ^a^	76 ± 28	112 ± 19 ^a^
Serum Vitamin B12 (pmol/L)	395 ± 360	1089 ± 420 ^a^	383 ± 297	1090 ± 832 ^a^
AA:EPA	18.9 ± 7.8	8.9 ± 5.0 ^b,c^	15.4 ± 7.7	8.9 ± 4.6 ^b^
TSL Depression	7.6 ± 2.4	3.7 ± 1.8 ^b,c^	3.4 ± 2.4	2.9 ± 1.2 ^b^
TSL Anxiety	7.9 ± 2.3	3.9 ± 1.8 ^b,c^	3.7 ± 2.4	3.2 ± 1.2 ^b^
TSL Mental Health Score	7.2 ± 1.9	3.6 ± 1.6 ^b,c^	3.4 ± 2.0	2.9 ± 1.0 ^b^

^a^ Indicates significant difference from baseline within group, paired-t; ^b^ Indicates significant difference from baseline within group, WSRT; ^c^ Indicates a significant difference between groups (Depressed/Anxious vs. Everyone Else), Mann–Whitney U.

**Table 7:** Magnitude of Effect of Nutritional Status on Targeted Symptoms List (TSL) Depression and Anxiety at one-year follow-up in Severely/Extremely Anxious/Depressed.

Effect Size Between Baseline and One-Year (95% CI)						
TSL Parameters	Low Vitamin B12 (<450 (pmol/L))	High Vitamin B12 (≥450 pmol/L)	Low Serum 25(OH)D <100 nmol/L	High Serum 25(OH)D ≥100 nmol/L	Combined Low B12 and 25(OH)D	Combined High B12 and 25(OH)D
**Severely/Extremely Anxious/Depressed**						
Depression	0.59 [−0.01–1.20]	1.95 [1.80–2.09]	0.80 [0.37–1.24]	2.01 [1.86–2.16]	0.61 [−0.13–1.34]	2.05 [1.90–2.20]
Anxiety	0.60 [−0.01–1.20]	2.04 [1.89–2.19]	0.65 [0.22–1.07]	2.13 [1.98–2.28]	0.66 [−0.08–1.39]	2.18 [2.03–2.33]
Mental score	0.57 [−0.05–1.18]	2.20 [2.05–2.35]	0.78 [0.35–1.22]	2.28 [2.12–2.44]	0.54 [−0.19–1.27]	2.33 [2.17–2.49]
*N*	24	539	47	516	15	510
**None/Slightly/Moderately Anxious/Depressed**						
Depression	0.06 [−0.04–0.16]	0.22 [0.19–0.25]	0.07 [−0.01–0.16]	0.23 [0.20–0.25]	0.04 [−0.09–0.17]	0.23 [0.20–0.26]
Anxiety	0.07 [−0.03–0.18]	0.23 [0.20–0.25]	0.11 [0.03–0.19]	0.23 [0.20–0.26]	0.05 [−0.08–0.18]	0.23 [0.21–0.26]
Mental score	0.10 [−0.01–0.20]	0.28 [0.26–0.31]	0.12 [0.04–0.21]	0.29 [0.26–0.32]	0.07 [−0.06–0.20]	0.29 [0.26–0.32]
*N*	730	10,303	1067	9966	452	9688

Cohen’s d (with confidence intervals) was used as a measure of effect sizes: 0.2 is small, 0.5 is medium, and 0.8 is large.

**Table 8:** Comparison of biomarkers of nutrition and mental health scores between case and control groups.

Parameter	Depressed/Anxious (Severe/Extreme) on No Medications (Cases, *n* = 107)		Depressed/Anxious on Medications (Controls, *n* = 214)		Changes from Baseline		*p* Value of Changes
	Baseline	One Year	Baseline	One Year	Cases	Controls	
Age (years)	47.2 ± 15	---	47.1 ± 15	---	---	---	
BMI	27.8 ± 6.5	27.3 ± 3.5	27.9 ± 6.0	27.8 ± 2.7	−0.54 ± 5.4	−0.10 ± 5.5	0.20
Serum 25(OH)D (nmol/L)	95 ± 49	130 ± 51 ^a^	72 ± 20	106 ± 37 ^a^	34 ± 45	34 ± 45	0.81
Serum Vitamin B12 (pmol/L)	441 ± 373	1061 ± 263 ^a^	379 ± 399	1080 ± 393 ^a^	619 ± 433	701 ± 548	0.06
AA:EPA	18.4 ± 6.2	8.9 ± 5.4 ^b^	18.7 ± 8.5	10.5 ± 2.7 ^b^	−9.5 ± 6.2	−8.2 ± 6.8	0.76
TSL Depression	7.6 ± 2.5	3.6 ± 1.7 ^b,c^	8.2 ± 1.9	3.9 ± 2.1 ^b^	−4.0 ± 2.7	−4.4 ± 2.6	0.36
TSL Anxiety	8.2 ± 2.0	4.0 ± 1.8 ^b,c^	8.0 ± 2.2	4.1 ± 1.9 ^b^	−4.1 ± 2.6	−3.9 ± 2.7	0.66
TSL Mental Health	7.4 ± 1.9	3.6 ± 1.5 ^b,c^	7.5 ± 1.7	3.8 ± 1.8 ^b^	−3.8 ± 2.2	−3.7 ± 2.3	0.96

^a^ Indicates significant difference from baseline within group, paired-t; ^b^ Indicates significant difference from baseline within group, WSRT; ^c^ Indicates a significant difference between groups (Depressed/Anxious and no meds vs. Depressed/Anxious on meds), Mann–Whitney U.

**Table 9:** Binary Logistic Regression of influencing factors on Targeted Symptoms List (TSL) mental parameters over one year.

TSL Parameter Improvement	Factor	Exp (B)	*p* Value	95% CI
**Anxiety (*R*^2^ = 0.05, *p* < 0.001)**	Age	0.99	0.90	0.977–1.021
	Gender (Male)	0.91	0.75	0.526–1.597
	Serum vitamin B12 (BL ≥ 450 pmol/L and FL ≥ 450 pmol/L)	Reference		
	Serum vitamin B12 (BL < 450 and FL ≥ 450 pmol/L)	0.83	0.54	0.476–1.475
	Serum 25(OH)D (BL ≥ 100 and FL ≥ 100 nmol/L)	Reference	0.25	
	Serum 25(OH)D (BL < 75 and FL ≥ 100)	1.71	0.10	0.950–3.455
	Serum 25(OH)D (BL 75–100 and FL ≥ 100)	1.03	0.92	0.564–1.872
	BMI, kg/m^2^	0.98	0.70	0.522–1.546
	Tobacco use (Never)	Reference	0.53	
	Tobacco use (Quit smoking)	0.89	0.70	0.522–1.546
	Tobacco use (current smoker)	0.51	0.26	0.156–1.668
	Alcohol use (Never)	Reference	0.61	
	Alcohol use (Light drinker)	0.73	0.32	0.392–1.369
	Alcohol use (Heavy drinker)	0.63	0.62	0.096–4.120
	Physical activity (No)	Reference	0.74	
	Physical activity (Mild)	1.49	0.28	0.710–3.155
	Physical activity (Moderate)	1.31	0.51	0.577–2.989
	Physical activity (Strenuous)	1.16	0.79	0.386–3.486
	Inflammation (CRP ≥ 10 mg/L)	0.25	0.18	0.032–1.936
	AA:EPA	0.98	0.30	0.951–1.015
**Depression (*R*^2^ = 0.1, *p* < 0.001)**	Age	0.98	0.22	0.961–1.009
	Gender (Male)	0.88	0.66	0.490–1.581
	Serum vitamin B12 (BL ≥ 450 pmol/L and FL ≥ 450 pmol/L)	Reference		
	Serum vitamin B12 (BL < 450 and FL ≥ 450 pmol/L)	0.77	0.41	0.428–1.418
	Serum 25(OH)D (BL ≥ 100 and FL ≥ 100 nmol/L)	Reference	0.15	
	Serum 25(OH)D (BL < 75 and FL ≥ 100) *	1.96	0.05	1.002–4.106
	Serum 25(OH)D (BL 75–100 and FL ≥ 100)	1.57	0.16	0.836–2.955
	BMI, kg/m^2^	0.98	0.65	0.940–1.040
	Tobacco use (Never)	Reference	0.59	
	Tobacco use (Quit smoking)	1.17	0.59	0.652–2.112
	Tobacco use (current smoker)	0.65	0.46	0.203–2.064
	Alcohol use (Never)	Reference	0.42	
	Alcohol use (Light drinker)	1.49	0.20	0.808–2.755
	Alcohol use (Heavy drinker)	1.04	0.96	0.169–6.474
	Physical activity (No)	Reference	0.47	
	Physical activity (Mild)	1.86	0.11	0.857–4.039
	Physical activity (Moderate)	1.63	0.26	0.695–3.823
	Physical activity (Strenuous)	1.80	0.34	0.535–6.060
	Inflammation (CRP ≥ 10 mg/L)	0.37	0.13	0.102–1.349
	AA:EPA	0.99	0.74	0.961–1.029
**Mental score (*R*^2^ = 0.1, *p* < 0.001)**	Age	0.98	0.11	0.957–1.005
	Gender (Male)	0.83	0.55	0.472–1.493
	Serum vitamin B12 (BL ≥ 450 pmol/L and FL ≥ 450 pmol/L)	Reference		
	Serum vitamin B12 (BL < 450 and FL ≥ 450 pmol/L)	0.84	0.58	0.474–1.516
	Serum 25(OH)D (BL ≥ 100 and FL ≥ 100 nmol/L)	Reference	0.29	
	Serum 25(OH)D (BL < 75 and FL ≥ 100)	1.76	0.10	0.960–3.595
	Serum 25(OH)D (BL 75–100 and FL ≥ 100)	1.18	0.59	0.639–2.192
	BMI, kg/m^2^	1.02	0.55	0.965–1.068
	Tobacco use (Never)	Reference	0.67	
	Tobacco use (Quit smoking)	1.03	0.91	0.586–1.811
	Tobacco use (current smoker)	0.59	0.39	0.170–2.023
	Alcohol use (Never)	Reference	0.75	
	Alcohol use (Light drinker)	1.11	0.73	0.602–2.053
	Alcohol use (Heavy drinker)	0.59	0.58	0.086–4.01
	Physical activity (No)	Reference	0.05	
	Physical activity (Mild) *	2.46	0.02	1.155–5.23
	Physical activity (Moderate) *	2.26	0.05	1.002–5.27
	Physical activity (Strenuous) *	4.99	0.01	1.317–18.96
	Inflammation (CRP ≥ 10 mg/L)	0.73	0.59	0.227–2.349
	AA:EPA *	0.98	0.03	0.956–0.998

* Significant predictors, BL = baseline, FL = Follow-up, BMI = Body Mass Index, Fruit, vegetable, and fish consumption were removed from model because of almost zero effect size and for simplicity.

## Supplementary Materials

The following are available online at http://www.mdpi.com/2072-6643/10/2/152/s1, Table S1: Comparison of Multivitamin/mineral formulations: Vital 2 Platinum vs. EMPowerplus Table S2: Lifestyle characteristics of population at baseline according to the reported levels of anxiety/depression, Table S3: Comparison of change in reported level of depression and anxiety at one year between case and control groups.
