# Supplementary material for: Database Analysis of Depression and Anxiety in a Community Sample—Response to a Micronutrient Intervention
Source: Nutrients. 2018 Jan 30;10(2):152. doi: 10.3390/nu10020152 (PMC5852728; doi:10.3390/nu10020152)
Supplement: Supplementary file 1 [file nutrients-10-00152-s001.docx]

**Table S1**. Comparison of Multivitamin/mineral formulations: Vital 2 Platinum vs. EMPowerplus

| **Nutrients** | **Vital 2 Platinum (per tablet)** | **True Hope Empowerplus (per capsule)** |
| --- | --- | --- |
| Vitamin A | 375 IU | 384 IU |
| Beta Carotene | 680 mcg | No |
| Vitamin B1 | 25 mg (thiamine HCl) | 1.2 mg (thiamin mononitrate) |
| Benfotiamine | 6 mg | No |
| Vitamin B2 | (riboflavin 5-phosphate) 2.5 mg | (riboflavin) 0.9 mg |
| Vitamin B3 (niacin) | 20.6 mg | No |
| Vitamin B3 (niacinamide) | 33.3 mg | 6 mg |
| Vitamin 5 (calcium-d-pantothenate) | 25 mg | 1.4 mg |
| Vitamin B6 (pyridoxal 5-phosphate) | 5 mg | 2.4 mg |
| Vitamin H | (Biotin) 75 mcg | (d-biotin) 72 mcg |
| Vitamin B9 (5-Methltertrahydrofolic acid) (folic acid) | 100 mcg | 96 mcg |
| Vitamin B12 | (methylcobalamin) 750 mcg | (cyanocobalamin) 60 mcg |
| Vitamin C | 125 mg | (ascorbic acid) 40 mg |
| Vitamin D3 (cholecalciferol) | 25 mcg (1000 IU) | 2.4 mcg (96 IU) |
| Vitamin E (Mixed tocopherols) | 25 mg | No |
| Vitamin E (Mixed tocotrienols) | 2.5 mg | No |
| Vitamin E (as d-alpha tocopheryl succinate) | No | 16 mg (24 IU) |
| Vitamin K2 | 45 mcg | No |
| Choline (bitartrate) | 25 mg | 36 mg |
| Inositol | 12.5 mg | No |
| Calcium | (HVP*chelate) 31.25 mg | (calcium phosphate dibasic) 88 mg |
| Magnesium | (HVP*chelate) 75 mg | (magnesium oxide) 40 mg |
| Manganese | (citrate) 0.42 mg | (manganese carbonate) 0.64 mg |
| Zinc (citrate) | 8 mg | (zinc oxide) 3.2 mg |
| Selenium | (selenomethionine) 50 mcg | (sodium selenite) 13.6 mcg |
| Copper | (HVP*chelate) 0.22 mg | (copper sulphate) 0.48 mg |
| Iodine (Ascophyllum nodosum)(whole plant) | 37.5 mcg | (lamina digitate/ascophylum nodosum) 13.6 mcg |
| Iodine (potassium iodide) | 37.5 mcg | No |
| Chromium | (polynicotinate) 125 mcg | (chromium chloride) 41.6 mcg |
| Molybdenum | (citrate) 100 mcg | (sodium molybdate) 9.6 mcg |
| Vanadium | (citrate) 10 mcg | (sodium metavanadate) 79.6 mcg |
| Boron | (citrate) 41.7 mcg | (boric acid) 28 mcg |
| Curcumin phytome extract | 10 mg | No |
| Rosemary Extract | 2.5 mg | No |
| Bilberry extract | 12.5 mg | No |
| Lutein | 1.5 mg | No |
| Zeaxanthin | 0.5 mg | No |
| LYC-O-MATO | 0.33 mg | No |
| Ubiquinol | 12.5 mg | No |
| Alpha-lipoic acid | 75 mg | No |
| Hesperidin | 4.2 mg | No |
| N-acetyl-L-cysteine | 62.5 mg | No |
| Iron (iron fumarate) | No | 0.9 mg |
| Phosphorus (from calcium phosphate dibasic) | No | 56 mg |
| Potassium (potassium chloride) | No | 16 mg |
| DL-Phenylanlanine | No | 24 mg |
| L-Glutamine | No | 12 mg |
| Citrus bioflavonoids | No | 16 mg |
| Grape seed extract | No | 3 mg |
| Myo-inositol | No | 12 mg |
| Gingko biloba | No | 2.4 mg |
| L-Methionine | No | 4 mg |
| Germanium sesquioxide | No | 1.38 mg |
| Nickel (nickel sulphate) | No | 1.96 mcg |

**Table S2.** Lifestyle characteristics of population at baseline according to the reported levels of anxiety/depression

| **Level of Anxiety or Depression Reported on EQ-5D** | **Fruit consumption (serving/day)** | | | | **Vegetable consumption (serving/day)** | | | | **Fish/tuna consumption (serving/week)** | | | **Tobacco use** | | | **Alcohol use** | | | **Physical activity** | | | |
| --- | --- | --- | --- | --- | --- | --- | --- | --- | --- | --- | --- | --- | --- | --- | --- | --- | --- | --- | --- | --- | --- |
|  | No | 1-2 | 3-4 | 5 & more | No | 1-2 | 3-4 | 5 & more | No | 1-2 | 3 & more | Never | Quit | Smoking | Never | Light drinker | Heavy drinker | No | Mild | Moderate | strenuous |
| None | 60 (32) | 975 (40) | 807 (42) | 260 (46) | 14 (24) | 764 (40) | 881 (42) | 443 (44) | 288 (39) | 831 (41) | 553 (44) | 3,130 (48) | 1,274 (39) | 865 (29) | 568 (43) | 1,982 (39) | 255 (25) | 341 (32) | 732 (37) | 804 (41) | 306 (44) |
| Slight | 67 (34) | 909 (37) | 689 (37) | 199 (35) | 22 (38) | 667 (34) | 794 (37) | 390 (39) | 263 (36) | 756 (37) | 463 (37) | 2,199 (33) | 1,243 (38) | 1,021 (35) | 445 (34) | 1,909 (38) | 341 (33) | 360 (34) | 727 (37) | 740 (38) | 253 (37) |
| Moderate | 41 (22) | 446 (18) | 312 (17) | 90 (16) | 13 (22) | 369 (19) | 364 (17) | 144 (14) | 131 (18) | 370 (18) | 203 (16) | 1,026 (16) | 617 (19) | 693 (24) | 231 (17) | 934 (19) | 305 (30) | 248 (24) | 404 (20) | 346 (18) | 107 (16) |
| **Severe** | **18 (10)** | **87 (4)** | **50 (3)** | **9 (2)** | **7 (13)** | **89 (5)** | **53 (3)** | **15 (2)** | **42 (6)** | **60 (3)** | **29 (2)** | **180 (3)** | **104 (3)** | **220 (8)** | **56 (4)** | **159 (3)** | **84 (8)** | **77 (7)** | **86 (4)** | **55 (3)** | **15 (2)** |
| **Extreme** | **3 (2)** | **31 (1)** | **9 (1)** | **4 (1)** | **2 (3)** | **31 (2)** | **11 (1)** | **6 (1)** | **8 (1)** | **20 (1)** | **12 (1)** | **60 (1)** | **47 (1)** | **103 (4)** | **25 (2)** | **56 (1)** | **38 (4)** | **27 (3)** | **31 (2)** | **21 (1)** | **8 (1)** |
| Total | 189 | 2,448 | 1,867 | 562 | 58 | 1,920 | 2,103 | 998 | 732 | 2,037 | 1,260 | 6,595 | 3,285 | 2,902 | 1,325 | 5,040 | 1,023 | 1,053 | 1,980 | 1,966 | 689 |
| Chi Square | P <0.001 | | | | P<0.001 | | | | P=0.003 | | | P<0.001 | | | P<0.001 | | | P<0.001 | | | |

**Table S3**. Comparison of change in reported level of depression and anxiety at one year between case and control groups

| **Baseline Level of Depression/Anxiety** | **Change at One Year among cases**  **Number (%)** | | | | **Change at One Year among controls**  **Number (%)** | | | |
| --- | --- | --- | --- | --- | --- | --- | --- | --- |
|  | **Worse** | **Improve** | **No Change** | **Total** | **Worse** | **Improve** | **No Change** | **Total** |
| Severe | 0 | 73 (94.8) | 4 (5.2) | 77 | 0 | 142 (97.3) | 4 (2.7) | 146 |
| Extreme | 0 | 29 (96.7) | 1 (3.3) | 30 | 0 | 67 (98.5) | 1 (1.5) | 68 |
| Total | 0 | 102 | 5 | 107 | 0 | 209 | 5 | 214 |
| Chi-Square (difference between groups | P=0.25 | | | | | | | |
